# Passively administered fluoxetine reaches the juvenile brain of FSL rats and reduces antioxidant defences, without altering serotonin turnover

**DOI:** 10.1186/s40360-024-00775-1

**Published:** 2024-08-16

**Authors:** Stephan F. Steyn, Malie Rheeders, Francois P. Viljoen, Linda Brand

**Affiliations:** Centre of Excellence for Pharmaceutical Sciences, Faculty of Health Sciences, North-West University, Hoffman Street, Potchefstroom, 2531 South Africa

**Keywords:** Breast milk, Flinders sensitive line, Norfluoxetine, Redox state, Serotonin turnover

## Abstract

**Background:**

Fluoxetine is present in breast milk, yet it is unclear to what extent it, or its active metabolite, norfluoxetine, reaches the brain of the infant and what the effects of such exposure on neurobiological processes are. We therefore aimed to quantify the concentration of passively administered fluoxetine and norfluoxetine in the whole brains of exposed Flinders sensitive line (FSL) offspring and establish their influence on serotonergic function and redox status.

**Methods:**

Adult FSL dams received fluoxetine (10 mg/kg/day), or placebo for fourteen days, beginning on postpartum day 04. Offspring were passively exposed to fluoxetine until postnatal day 18 and euthanized on postnatal day 22. Whole brain fluoxetine, norfluoxetine, serotonin (5-HT), 5-hydroxyindoleacetic acid (5-HIAA), and reduced (GSH) and oxidized glutathione (GSSG) concentrations were measured via liquid chromatography–mass spectrometry (LC-MS) analysis.

**Results:**

Whole-brain serotonin and 5-hydroxyindoleacetic acid concentrations, and serotonin turnover (5-HIAA/5-HT) were comparable between strains. Treatment-naïve FSL rats had lower GSH and higher GSSG whole-brain concentrations, relative to FRL controls, and an overall decreased GSH/GSSG ratio. Passively administered fluoxetine resulted in undetectable whole-brain concentrations, while norfluoxetine averaged 41.28 ± 6.47 ng/g. Serotonin turnover of FSL rats was unaffected by passively administered fluoxetine, while redox status (GSH/GSSG) was decreased.

**Conclusion:**

Our findings confirm that passively administered fluoxetine reaches the infant brain in the form of norfluoxetine and may manipulate processes of oxidative stress regulation. Further studies into the long-term bio-behavioural effects are however needed to effectively inform breast feeding mothers on the safety of antidepressant-use.

**Supplementary Information:**

The online version contains supplementary material available at 10.1186/s40360-024-00775-1.

## Background

Post-COVID-19 prevalence of maternal psychological stress has significantly increased to such an extent that as much as 36% of mothers with children younger than 18 months are reported to struggle with depression and anxiety symptoms [1]. The global prevalence of postpartum depression, regardless of age, giving birth for the first time (primiparous), or being a single mother, is estimated to be between 14 [2] and 17% [3]. And although the prevalence appears to peak at twelve months postpartum, this is statistically comparable to earlier time points (i.e., 0, 3 and 6 months) [3].

Neonaticide and infanticide is unfortunately often associated with postpartum depression yet is one of the worst documented causes of death, resulting in systematic data on this topic to be scarce [4]. Early records do however suggest that an infant under the age of one, is killed daily in the United States, with this number considered to be a significant underestimation of the current global incidence [5]. Other consequences of postpartum depression, as reviewed and extensively discussed by Mokwena [6], include difficulty breastfeeding, which can augment the depressive symptoms by fuelling feelings (or perceptions) of being a “failed mother” [7]. Increased risk for child malnutrition, which can in turn adversely influence general health and childhood development is also related to postpartum depression, as are compromised emotional attachment to the child [8]. Social and academic development of the child is also negatively affected by postpartum depression and can increase the risk for the development of mental health conditions (juvenile depression) and impaired cognitive development [8]. Alarmingly, these depressive symptoms (and their consequences) can last up to eleven years post-pregnancy, and negatively affect the immediate and broader family [8].

It is therefore worth noting that the first pharmacological treatment option for postpartum depression to receive FDA-approval, was brexanolone (a gamma-aminobutyric acid A (GABA) receptor positive allosteric modulator) [9]. Yet, being only available in a continuous intravenous infusion dosing form, its use was limited, for practical and financial reasons. In 2023, however, zuranolone, another neuroactive steroid that increases GABA release by modulating GABAergic receptors, was approved as the first oral dosage form for treating postpartum depression, at a suggested daily dose of 50 mg for fourteen days [10]. It is noteworthy that although no official guidelines exist for postpartum depression, selective serotonin re-uptake inhibiting (SSRI) drugs, including fluoxetine, are generally recommended by clinicians [11, 12]. In fact, according to meta-analyses, SSRI drugs are at least superior to placebo controls for the treatment of postpartum depression and are also well tolerated [13, 14]. The consensus, however, is that these studies are generally small and heterogeneous, leading to low certainty of evidence. Either way, because fluoxetine crosses the placenta and is present in breast milk [15], it could potentially pose a health risk to the infant [15], often resulting in pregnant and breastfeeding women being sceptical to use antidepressants. Importantly, refusal of (pharmacological) antidepressant treatment can negatively influence the development of a child, as postpartum depression can lead to maternal neglect [16], which could set off a range of adverse and fatal consequences.

Fluoxetine increases serotonin levels by inhibiting the serotonin transporter, thereby preventing the re-uptake of serotonin into the neuron [17]. The active metabolite, norfluoxetine, has an elimination half-life three times longer than that of the parent compound, resulting in prolonged pharmacodynamic effects, even after cessation of treatment [17]. Secondary to the serotonergic-enhancing effects, fluoxetine also affects mitochondrial function. This is of note as mitochondrial (dys)function has recently gained more attention as a promising and novel antidepressant target [18]. In healthy mitochondria, reactive oxygen species production is counterbalanced by mitochondrial-produced antioxidant defences [19], which prevents cellular damage and/or dysfunctional processes. Conversely, when mitochondria function sub-optimally, reactive oxygen species production is increased [20], unopposed by antioxidant defences, leading to cellular damage and apoptosis [21]. That fluoxetine induces both positive and negative bio-energetic effects [22], highlights its potential to influence neurodevelopmental processes, specifically during an energy dependent (i.e., early postpartum) period.

Clinical data regarding the neurochemical effects of passively administered fluoxetine (i.e., via breast milk) in offspring are largely limited, because of the practical and ethical implications. To this end, the Flinders sensitive line (FSL) rat is a validated rodent model of depression [23], displaying behaviour and neurochemical constructs akin to the clinical condition. That these discrepancies are successfully reversed by pharmacological [23] and non-pharmacological antidepressant interventions [24], makes it a translatable model to investigate the neurodevelopmental effects of passively administered fluoxetine.

The current study therefore set out to determine how much of the passively administered fluoxetine reaches the juvenile brain, and what the effects thereof are on serotonin turnover and redox state. We expect juvenile FSL rats to have higher serotonin turnover and decreased antioxidant defences, relative to Flinders resistant line (FRL) controls, and that passively administered fluoxetine will result in high norfluoxetine/fluoxetine whole brain values and reverse the mentioned serotonin and redox state profiles.

## Methods

### Study layout

**Fig. 1:**
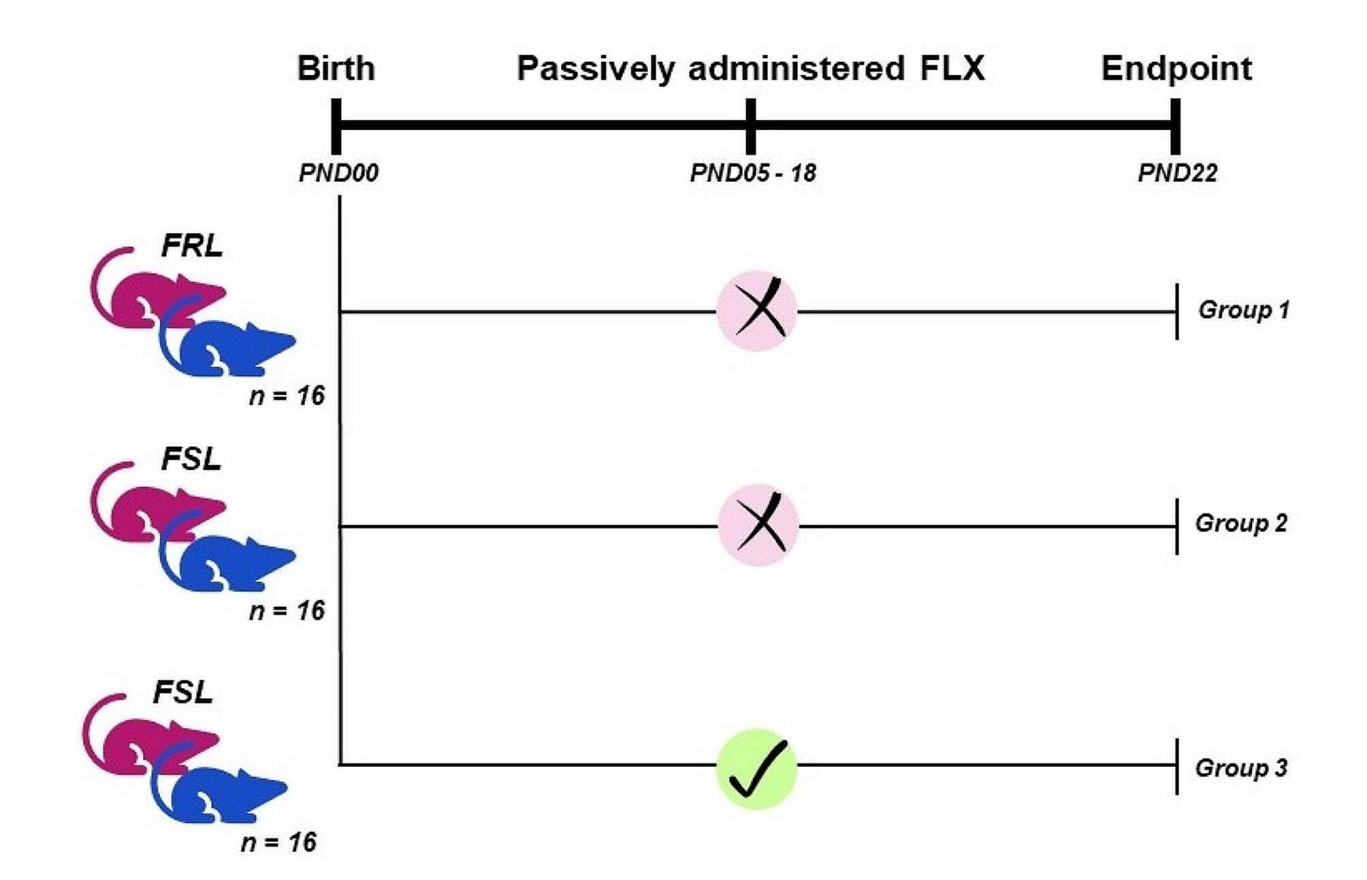
Graphical representation of the study layout. Male (blue) and female (pink) Flinders sensitive and resistant line pups were either exposed to passively administered fluoxetine during early-life development (i.e., PND05 to 18), or not. *FLX* Fluoxetine (10 mg/kg/day). *FRL* Flinders resistant line. *FSL* Flinders sensitive line. *PND* Postnatal day

In related, and unpublished work [25], female FSL rats were administered fluoxetine (10 mg/kg/day) [26, 27] or distilled water (vehicle control), subcutaneously, from postpartum day 04 (PPD04) until PPD18 (Fig. 1). On PND22, the dams (adult female rats that recently gave birth) and the pups (juvenile rodent offspring of these dams) were euthanized, via decapitation. Animals were euthanized without anaesthesia to eliminate any confounding influence on the neuro- and/or biochemical functions, or anatomical integrity [28] of the collected samples. Moreover, the inclusion of anaesthesia has also been reported to negatively affect the comparability of research findings, across studies [28]. Consequently, euthanasia via decapitation, without anaesthesia, is indeed an accepted method [29], with evidence supporting it to lead to “prompt, painless unconsciousness in laboratory rodents” [30]. Whole brains of the pups were removed, snap frozen in liquid nitrogen and stored at -80 °C until analyses could be performed. Ethical approval was given for the collection and analyses of these samples from the mentioned unpublished work [25]. For the FRL group, postpartum dams received vehicle control (distilled water) during the same intervention period [25].

### Tissue collection, storage, and analysis

Each brain tissue sample was individually weighed prior to preparation. Hereafter, 500 µL of the sample preparation solution (0.1% formic acid in methanol, and acetonitrile; 1:1– for protein precipitation) was added to the specific sample, containing 200 ng/ml escitalopram, as by virtue of some structural similarities to fluoxetine, acted as the internal standard. The mixture was sonicated twice for 12 s, at 14 µ, whereafter another 500 µl of the preparation solution was added and left on ice for 20 min to complete protein precipitation and extraction of analytes. After samples were centrifuged at 20 817 rcf (relative centrifugal force, also known as g-force) for 20 min at 4 ºC, the samples (2 µL injection volume) were analysed, using a Venusil ASB C18 column, 2.1 × 150 mm, 3 μm on an Ultivo Triple Quadrupole LC-MS (liquid chromatography–mass spectrometry analysis), with multiple reaction monitoring (Table 1 details the instrument settings for the analysis). As summarized in Table 2, the gradient mobile phase (0.3 ml/min flowrate) consisted of A: 0.1% formic acid/HPLC grade water and B: 0.1% formic acid/acetonitrile. Agilent MassHunter^®^ software was used to control and run the analysis.

**Table 1:** Optimum instrument settings for the identification and quantification of product ions

**LC instrument settings**							
Flow rate				0.3 ml/min			
Injection volume				2 µl			
Run time				18 min (15 min plus 3 min post-time)			
**Mass spectrometer settings**							
Source parameter				Positive value			
Gas temperature				350 °C			
Gas flow				13 l/min			
Nebulizer				60			
Capillary voltage				4000 V			
**Analyte setup**							
**Analyte**	**Transition (m/z)**	**Dwell** **(ms)**	**Fragmentor** **(V)**	**Collision energy** **(V)**	**Polarity**	**Scan mode**	**RRT (min)**
5-HIAA	192.1 ≥ 145.9	100	61	17	Positive	MRM	± 8.446
5-HT	177.1 ≥ 160.1		56	13			± 2.196
FLX	310.1 ≥ 148.1		51	5			± 9.201
GSH	308.1 ≥ 179.0		76	9			± 1.702
GSSG	613.2 ≥ 231.0		121	37			± 1.692
nFLX	296.1 ≥ 74.0		51	169			± 9.144
I.Std (ESC)	325.2 ≥ 108.9		76	53			± 8.963

*5-HIAA* 5-hydroxyindoleacetic acid. *5-HT* 5-hydroxytryptamine. *ESC* Escitalopram (internal standard). *FLX* Fluoxetine. *GSH* Glutathione. *GSSG* glutathione disulphide. *MRM* multiple reaction monitoring. *nFLX* Norfluoxetine. *RRT* relative retention time

**Table 2:** Mobile phase gradient setup

Step	Time (min)	A (%) HPLC water / 0.1% FA	B (%) ACN / 0.1% FA
1	Start condition 0	95.0	5.0
2	3.0	95.0	5.0
3	4.3	0.0	100.0
4	12.0	0.0	100.0
5	14.0	95.0	5.0
6	15.0	95.0	5.0
7	Post-time 3	95.0	5.0

The mobile phase was made up of HPLC water, 0.1% (v/v) formic acid (FA) and 0.1% acetonitrile (ACN), with a 3-minute post-time running period included

### Power and statistical analysis

Statistical analyses were performed in IBM^®^ SPSS^®^ Statistics and Graphpad Prism^®^, with the initial power analysis performed in G*Power^®^. An A priori test, set at an effect size of 0.8, 0.05 α error probability, and 80% power, and Sensitivity analysis, justified the current group sizes. All data sets were screened for outliers (Grubbs’ test) and tested for normality of distribution (Shapiro-Wilk test). Independent *t*-tests with Welch correction (or Mann-Whitney *U*-test) were used, with a *p*-value < 0.05 (two-tailed) accepted as significant. The unbiased Cohen’s *d* value was calculated as effect magnitude and considered large when ≥ 0.8. To analyse the influence of sex, an ordinary two-way ANOVA (analysis of variance) considered sex and strain (or treatment), with the partial eta squared (*η*_*p*_^*2*^) representing the effect magnitude.

## Results

### Mass spectrometry results

**Fig. 2:**
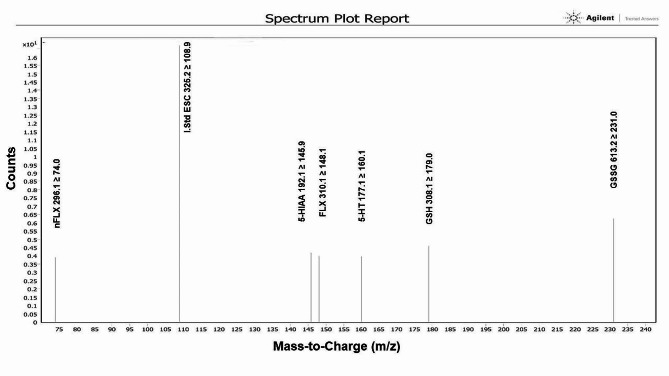
Mass spectrometry output spectra for the different analytes

Indicated in Fig. 2 are the various spectra results for the different analytes.

## Whole brain fluoxetine and norfluoxetine concentrations

Brain weight averaged 1021.0 ± 73.85 mg, with no statistical differences between the different experimental groups (*Strain*: *t*_25.0_ = 1.27, *p* = 0.21, *d*_*unb*_ = 0.4 [-0.3; 1.1]; *FLX*: *t*_30.0_ = 0.71, *p* = 0.48, *d*_*unb*_ = 0.2 [-0.4; 0.9]). Unfortunately, the pups were not weighed prior to being euthanized and therefore, brain weight could not be expressed as a percentage of body weight.

Although whole brain fluoxetine was undetectable (limit of detection was 7.81 ng/ml for both fluoxetine and norfluoxetine), norfluoxetine was successfully quantified, averaging 41.28 ± 6.47 ng/g in juvenile FSL rat brains, independent of sex (*t*_8.82_ = 0.73, *p* = 0.48, *d*_*unb*_ = 0.4 [-0.6; 1.4]).

### Serotonin turnover

**Fig. 3:**
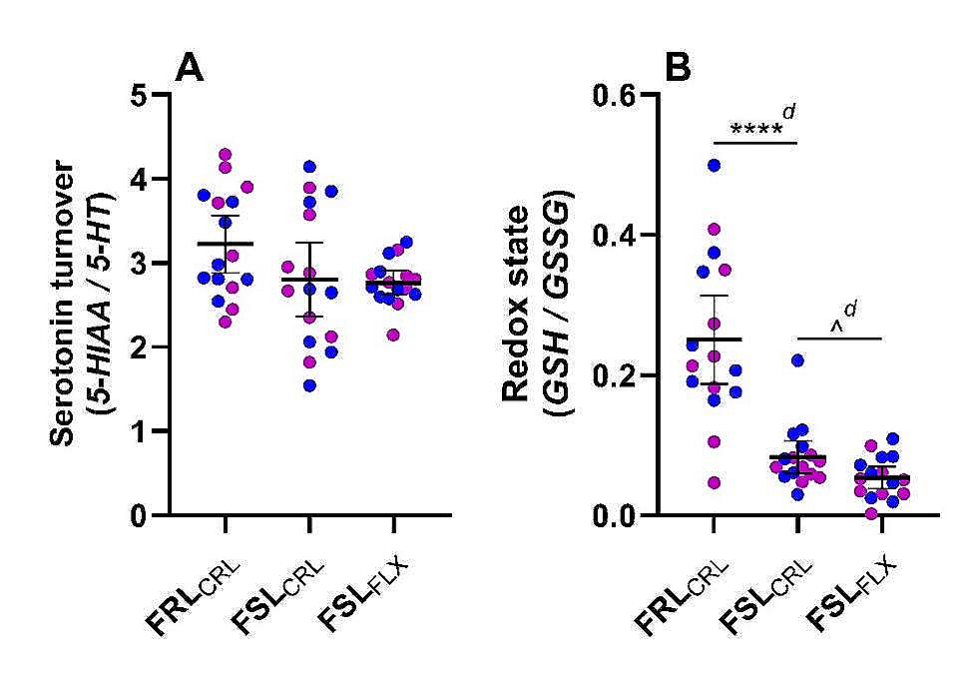
Whole brain serotonin turnover and redox state of FSL and FRL rats. Data represent the mean ± 95% CI, with male and female indicated in blue and purple, respectively, and **** *p* ≤ 0.0005, ^ *p* < 0.05, and d ≥ 0.8 (significant large effect) vs. indicated group. Redox state data of the FSL _*CRL*_group was not normally distributed, with one outlier identified but not removed. *5-HIAA* 5-hydroxyindoleacetic acid. *5-HT* 5-hydroxytryptamine. *CRL* Control. *FLX* Fluoxetine (10 mg/kg/day). *FRL* Flinders resistant line. *FSL* Flinders sensitive line. *GSH*: Glutathione. *GSSG* Glutathione disulphide

Serotonin turnover (5-HIAA/5-HT) was comparable between strains (Fig. 3A; *t*_28.3_ = 1.61, *p* = 0.12, *d*_*unb*_ = 0.6 [-0.1; 1.3]), with no statistically significant influence of passively administered fluoxetine in FSL rats (*t*_18.2_ = 0.18, *p* = 0.86, *d*_*unb*_ = 0.06 [-0.6; 0.8]). Sex again had no statistically significant influence on the serotonin turnover, either between strains (*F*_*1*, 28_ = 0.09, *p* = 0.77, *η*_*p*_^*2*^ = 0.003), or between FLX and CRL FSL offspring (*F*_1, 28_ = 0.08, *p* = 0.78, *η*_*p*_^*2*^ = 0.003).

As for the individual markers, both 5-HT and 5-HIAA (Table 3) were comparable between strain (5-HT: *t*_27.7_ = 0.47, *p* = 0.64, *d*_*unb*_ = 0.2 [-0.5; 0.9]; 5-HIAA: *t*_23.8_ = 1.23, *p* = 0.23, *d*_*unb*_ = 0.4 [-0.3; 1.1]) and treatment groups (5-HT: *t*_27.1_ = 0.48, *p* = 0.63, *d*_*unb*_ = 0.2 [-0.5; 0.9]; 5-HIAA: *t*_20.0_ = 0.10, *p* = 0.92, *d*_*unb*_ = 0.03 [-0.7; 0.7]) (Table 3).

**Table 3:** Summary of whole brain serotonin and redox markers in FSL and FRL juvenile rats

Neurochemical markers	FRL	FSL	
	control	control	passive fluoxetine
5-HIAA (ng/g)	105.2 ± 16.4	95.06 ± 28.81	95.88 ± 11.90
5-HT (ng/g)	33.28 ± 5.40	34.07 ± 4.01	34.67 ± 2.86
GSH (µg/g)	32.62 ± 7.07	25.54 ± 5.92^*a)*^	18.22 ± 6.75^*b)*^
GSSG (µg/g)	163.30 ± 77.44	338.80 ± 106.7^*a)*^	384.10 ± 141.00^*b)*^

Limits of detection were 3.90 ng/ml (5-HIAA and 5-HT), 0.31 µg/g (GSH) and 0.62 µg/g (GSSG). Final concentrations are expressed as w/w due to brain tissue being the sample matrix. The values used for calculations of the ratios are presented here as mean ± SD (*n* = 16 per group)

^*a)*^statistically different (*p* < 0.05), compared to FRL control group

^*b)*^statistically different (*p* < 0.05), compared to FSL fluoxetine group

*5-HIAA* 5-hydroxyindoleacetic acid. *5-HT* 5-hydroxytryptamine. *GSH* Glutathione. *GSSG* glutathione disulphide

### Redox state

Compared to FSL controls, the GSH/GSSG ratio was higher in juvenile FRL rats (Fig. 3B; *U* = 24, z = -3.92, *p* ≤ 0.0005, *d*_*unb*_ = 1.8 [1.0; 2.7]), with no significant sex differences (*F*_1, 28_ = 1.57, *p* = 0.22, *η*_*p*_^*2*^ = 0.05). Passively administered fluoxetine further decreased the GSH/GSSG ratio in FSL rats (*U* = 74, z = -2.04, *p* = 0.04, *d*_*unb*_ = 0.8 [0.05; 1.5]), also independent of sex (*Treatment: F*_1, 28_ = 0.25, *p* = 0.08, *η*_*p*_^*2*^ = 0.11).

As summarized in Table 3, the GSH concentrations of treatment-naïve FRL pups were higher than their FSL control counterparts (*t*_29.1_ = 3.07, *p* = 005, *d*_*unb*_ = 1.1 [0.3; 1.8]), which in turn was higher than that of FLX-exposed pups (*t*_29.5_ = 3.27, *p* = 0.003, *d*_*unb*_ = 1.1 [0.4; 1.9]). Interestingly, although the mean GSSG concentration of FSL controls were higher than that of FRL controls (*t*_27.4_ = 5.33, *p* ≤ 0.0005, *d*_*unb*_ = 1.8 [1.0; 2.7]), it was statistically comparable to that of FLX-exposed pups (*t*_27.9_ = 1.02, *p* = 0.32, *d*_*unb*_ = 0.4 [0.3; 1.1]) (Table 3).

## Discussion

### Neurochemical construct of the FSL rat

The juvenile FSL rat has been described as a suitable rodent model for childhood depression [31]. Although we previously reported increased hippocampal serotonin turnover in PND38 FSL rats [32], no strain differences were observed in whole brains on PND22 in the current study. This is in accordance with an earlier observation in the nucleus accumbens of FSL and Sprague-Dawley juvenile rats [33]. Age-dependent differences were, however, noted here [33], suggesting developmental monoaminergic changes to influence the behavioural profile of these animals. This hypothesis warrants further investigation, as the exact role of serotonin in the pathophysiology of depression has been questioned [34] and recently become a topic of debate [35, 36].

Glutathione (GSH) is a potent intra- and extracellular antioxidant with reduced defensive protection when oxidized to glutathione disulphide (GSSG). A lower GSH/GSSG value therefore indicates compromised antioxidant defences, and infers increased oxidative stress [37]. In line with our previous findings, the current results suggest that pre-pubertal FSL rats have reduced antioxidant defences, which may contribute to brain atrophy [32]. The current findings therefore support the neurochemical construct of the FSL rat as a suitable model for childhood depression, appropriate to investigate the neurotropic effects of passively administered fluoxetine in the developing brain.

### The fluoxetine effect

That the serotonergic pathway matures before the adrenergic one [38], could sensitize the developing brain to the effects of increased serotonin, caused by passively administered fluoxetine. In the current study, we quantified whole brain fluoxetine and norfluoxetine levels, which to the best of our knowledge is novel. Unfortunately, fluoxetine was undetectable, whilst the mean norfluoxetine concentration measured 41.28 ± 6.47 ng/g in FSL pups. To determine whether the measured norfluoxetine concentration is indeed realistic, certain literature assumptions had to be considered, and study limitations, accepted. First, according to Caccia and colleagues [39], the levels at 3 h were 2.4 (742 ng/g) ± 0.5 and 2.5 (738 ng/g) ± 0.5 nmol/g respectively for fluoxetine and norfluoxetine, with no preference for concentrating in the different rodent brain regions, following a single 10 mg/kg oral dose. After 30 h, these levels decreased to 0.02 (14.84 ng/g) ± 0.01 fluoxetine and 1.1 (324.83 ng/g) ± 0.5 nmol/g norfluoxetine [39]. If these values are extrapolated to 96 h (the four-day washout period of the current study) and converted to ng/g, a level of 80 ng/g norfluoxetine could be expected from a 10 mg dose. A concentration of 31.7 ng/g will therefore be expected of a 3.95 mg dose which is equivalent to the concentration of 41.28 ng/g in this study. Second, because fluoxetine concentration was not measured in the breast milk, it is not possible to determine the concentration pups were exposed too. However, available data [40–45] suggest that a mean value of 3.96 ± 2.25% of the maternal weight-adjusted fluoxetine dose is expressed in the breast milk, which may have been true in the current study. These calculations therefore align our findings with those of others, although an increase in plasma norfluoxetine/fluoxetine ratio in infants exposed to passively administered fluoxetine over time has been reported [41, 46, 47]. Caccia and colleagues [39] further reported norfluoxetine/fluoxetine ratio to increase from 1.0 to 54 in rodents, 30 h after a single fluoxetine dose, which would translate to 0.76 ng/ml fluoxetine after 90 h (below the limit of detection in the current study). The shorter half-life of fluoxetine and the fact that some pups were already dependent on solid food, and therefore consumed less (if any) breast milk (and fluoxetine) by PND22 could also explain why we were unable to detect fluoxetine in juvenile brains. Nevertheless, considering the serotonergic-enhancing potency of norfluoxetine [41], serotonergic turnover may have been affected.

Whole brain serotonin turnover of juvenile FSL rats, were however unaffected by passively administered fluoxetine. Although unexpected, this result is in line with another study [46] reporting corresponding baseline and post-exposure plasma serotonin levels in the babies of breastfeeding, fluoxetine-treated mothers. More recently, de Andrade Silva and colleagues [48], reported that 21-days of 10 mg/kg of subcutaneous fluoxetine did not induce any serotonin transporter or receptor expression differences in the hippocampi of juvenile Wistar rats. Together with the current findings, reports of unaltered behaviour in Wistar offspring, exposed to escalating doses of passively administered fluoxetine [49] further support a lack of effects of passively administered fluoxetine on serotonergic transmission.

As for the redox state effect, passively administered fluoxetine further compromised whole brain antioxidant defences in FSL pups, aligning with reports of decreased hippocampal GSH/GSSG ratios and glutathione *S*-transferase [48]. Interesting however, is that hippocampal malondialdehyde, superoxide dismutase and brain-derived neurotrophic factor were unaffected, whereas the NAD/NADH ratio was increased in these pups [48]– altogether suggesting decreased oxidative stress damage, and improved mitochondrial function. The possibility exists that the fluoxetine-induced inhibition of glutathione reductase, and consequent compromised antioxidant defences and increase oxidative stress, could be beneficial, for example, in combating intracellular abnormalities, such as tumour cells [50]. That no evidence of increased oxidative stress damage were observed in a similar investigation [48], could either be explained by compensatory mitochondrial mechanisms (i.e., increased NAD/NADH), and/or by the mitochondrial altering effects, induced by fluoxetine [22]. Either way, these observations point towards a more complex redox state effect of passively administered fluoxetine that warrants further investigation. Moreover, whether these neurochemical effects would benefit or impair behaviour in these juveniles, remains unclear.

## Conclusion

We quantified whole brain norfluoxetine concentrations of passively administered fluoxetine and evaluated the serotonergic and redox state effect in a pre-pubertal rodent model of depression. Our findings confirm that passively administered fluoxetine reaches the juvenile brain in the form of norfluoxetine, without altering serotonin turnover, and may manipulate processes of oxidative stress regulation. As to the nature of this manipulation, further studies are required to determine whether the observed decrease in antioxidant defences adversely influence juvenile behaviour. Further studies into the long-term bio-behavioural effects are needed to effectively inform breastfeeding mothers on the safety of antidepressant-use.

### Electronic supplementary material

Below is the link to the electronic supplementary material.

Supplementary Material 1

## Data Availability

No datasets were generated or analysed during the current study.

## Abbreviations

5-HIAA: 5-hydroxyindoleacetic acid
5-HT: 5-hydroxytryptamine (serotonin)
FLX: Fluoxetine
FRL: Flinders resistant line
FSL: Flinders sensitive line
GABA: Gamma-aminobutyric acid A
GSH: Glutathione
GSSG: Glutathione disulphide
nFLX: Norfluoxetine
PND: Postnatal day
PPD: Postpartum day
SSRI: Selective serotonin re-uptake inhibitor
