# Supplementary figures and images for: Passively administered fluoxetine reaches the juvenile brain of FSL rats and reduces antioxidant defences, without altering serotonin turnover
Source: BMC Pharmacol Toxicol. 2024 Aug 16;25:51. doi: 10.1186/s40360-024-00775-1 (PMC11330128; doi:10.1186/s40360-024-00775-1)

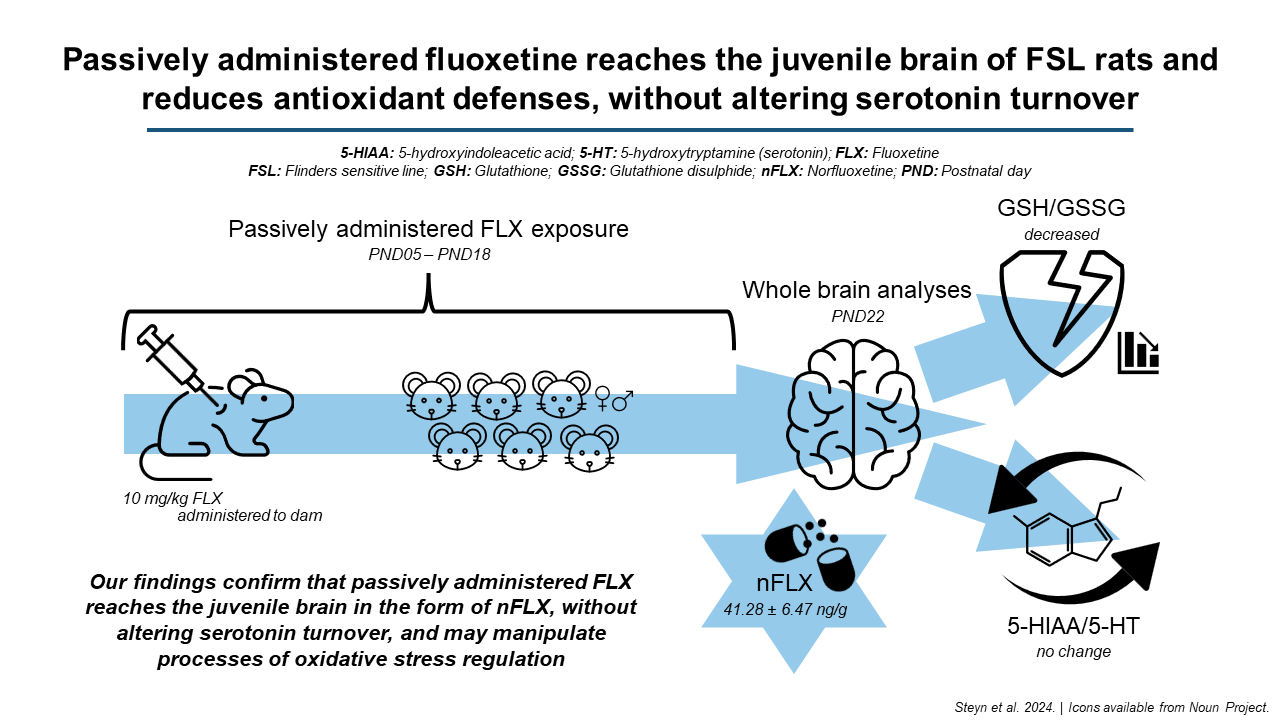

Supplement: Supplementary file 1 — Supplementary Material 1 [file 40360_2024_775_MOESM1_ESM.tif]
